# Integrating intercultural activities into teaching Mandarin for international students in China

**DOI:** 10.3389/fpsyg.2022.972733

**Published:** 2022-11-10

**Authors:** Na Liu

**Affiliations:** School of Foreign Languages, Jinchu University of Technology, Jingmen, China

**Keywords:** intercultural activities, Mandarin, international students, intercultural communicative competence, teaching

## Abstract

This study explores the intercultural activities in teaching Mandarin to international students in China. A sample of five Mandarin teachers with rich experience in teaching international students within Chinese universities. Data were collected through classroom observation, teachers’ interviews and reflective journals and analyzed through thematic analysis using the framework of Byram’s notion of intercultural competence in language teaching and learning. The results show that intercultural activities can effectively promote Mandarin teaching for international students. The findings also suggest Mandarin teachers can provide more opportunities for international students to share their values and opinions and adopt more activities to teach cultural knowledge. Accordingly, recommendations on preparing intercultural competence are concluded and put forward, which might support future teaching.

## Introduction

Mandarin learning has been booming worldwide in the past few decades because of the increasing number of international students. With this boom, the subject of teaching “Chinese as a Second language” (CSL) has been gradually attracting scholars’ attention and has become a research hot spot, forming a new discipline (Dong, 2012). Mandarin classes for international students in China usually emphasize intensive training before the study to lay a foundation (Ji, 2016).

According to the previous research on Mandarin education for international students in China, different people may have different goals in studying in Chinese universities. Still, the primary educational expectations of international students in China are to learn Chinese, understand Chinese society and culture, and gain personal development opportunities (Ding, 2010; Liu et al., 2013; Yu and Cao, 2015). To assist international students in understanding and adapting to Chinese culture, it is of great necessity to develop students’ intercultural competence by implementing intercultural teaching and systematically incorporating Chinese culture content in Mandarin education. In other words, besides basic Mandarin teachings such as words, phrases, and grammar, Chinese culture teaching is an essential part of the classroom teaching for Chinese-learning international students in China (Ding, 2010; Huang and Chen, 2020).

Taking the Belt and Road Initiative as an opportunity, colleges carry out intercultural education for international students in Mandarin education to serve the national strategy. Liu and Liu (2020) concluded that Chinese cultural education falls into three categories: types of regional culture, the teaching of regional culture, and curriculum design. Feng and Shi (2019) researched talent-training modes for International Students in city-based and application-oriented universities. The importance of intercultural activities in language teaching is explicitly stated and highly valued for developing learners’ intercultural competence (Byram et al., 2002; Liddicoat, 2008; McHugh, 2012; Moeller and Nugent, 2014). However, most language researchers or teachers are concerned about how to foster intercultural competence of EFL learners in China, while the development of international CSL students’ intercultural competence has not received enough attention (Karabinar and Guler, 2012). Ding (2010) illustrated the kinds of education that international students needed. Dong (2012) conducted comprehensive research on international student education in China. Liu and Liu (2020) made a summary review of the studies on the classroom teaching of Chinese culture to improve international students’ intercultural competence. Nevertheless, there is very little evidence of the inclusion of intercultural activities in Mandarin teaching.

The present study attempted to investigate intercultural activities in Mandarin classes, teaching the international students from the lesson proper and activity period. This study focused on integrating intercultural activities in Mandarin teaching in China.

## Literature review

### Intercultural competence

The notion of intercultural competence can be traced back to Hymes’s theory of communicative competence (Hymes, 1972). It extends Chomsky’s linguistic competence as the ideal speaker/listener and emphasizes the aim of being an intercultural speaker mediating between different cultures. It considers real-life communication and requires individuals’ competence in a culture that empowers them to know what to say to whom and how to say it appropriately in a given situation (Gardner, 1985; Byram, 1997; Byram and Wagner, 2018).

Researchers have proposed various definitions and taxonomies of intercultural competence, such as intercultural communication competence, cultural competence, intercultural sensitivity and global competence (Bennett, 1991; Byram, 1997; Fantini, 2000; Spitzberg, 2000; Deardorff, 2006; Hunter et al., 2006; Barnett and Lee, 2007). The most widely known version of intercultural competence stems from the work of Byram. He makes a distinction between intercultural communicative competence and intercultural competence and insists that the former includes the latter. He defines intercultural communicative competence as the ability “to ensure a shared understanding by people of different social identities, and [the] ability to interact with people as complex human beings with multiple identities and their individuality” (p. 5). In his model, intercultural competence is composed of five elements: attitudes, knowledge, skills of interpreting and relating, skills of discovery and interaction, and critical cultural awareness (Byram, 1997; Byram et al., 2002). Chen and Starosta (1998) hold an opinion that intercultural competence as the ability to effectively and appropriately execute communication behaviors to elicit the desired response in a specific environment, encompasses three interdependent aspects, the affective perspective for developing intercultural sensitivity, the cognitive perspective for developing intercultural awareness, and the behavioral perspective for developing intercultural adroitness (Chen and Starosta, 1996; Gong et al., 2018). Fantini (2000) assumes the four dimensions of intercultural competence are knowledge, attitude, skills, and awareness. According to Wiseman (2002), the three vital ingredients to develop or accomplish intercultural competence are self-knowledge/awareness, experience and knowledge about a particular culture, and positive change or action for successful interaction with the identified culture. In other words, knowledge, motivation, and skills are essential requirements to interact effectively and appropriately with members of different cultures. Intercultural communicators require having a deep understanding of the similarities and differences in the way of thinking and living in other cultures, broadening horizons, and developing flexible communication skills that can adapt to various social and cultural environments (Beamer, 1992; Scarino, 2009).

### Intercultural activities in language teaching

Combining language teaching with intercultural activities in language classroom plays a critical role in cultivating language learners’ intercultural competence (Byram et al., 2002; Robatjazi, 2008; Barrett et al., 2014; Güven, 2015; Gu, 2016). Traditional language teaching has focused on improving language learners’ linguistic competence in the last two or three decades (Larzén, 2005). However, with the trend of globalization and the development of information technology, people from various regions and cultures are getting closer and having more interactions with each other in the global village. Thus, language educators have been involved in cultivating language learners’ intercultural competence and emphasizing the role of culture in language classrooms, which prepares them for interaction with people of other cultures (Byram, 1997; Aguilar, 2008; Irimia, 2012).

Culture is the central part of language learning, furthermore, intercultural teaching and learning process for international education, which needs to involve more than one language and culture (Crichton and Scarino, 2007). Intercultural activities integral to language teaching and learning focus on the relationship between languages and cultures (Byram et al., 2002; Larzén, 2005; Crichton and Scarino, 2007; Liddicoat and Scarino, 2013). The cultural “content” in intercultural teaching and learning is not just a body of cultural knowledge and information to be introduced and memorized; more importantly, it lies in comparing different cultures and analyzing and reflecting on how culture influences what people think, say, and do (Byram et al., 2002; Crichton and Scarino, 2007). Moreover, intercultural teaching encourages sharing knowledge and discussing values and opinions (Welikala and Chris, 2008; Svenja and Kerstin, 2019).

Integrating intercultural activities into language teaching may benefit those who interact with people of other cultures and help avoid the stereotyping that accompanies perceiving someone through a single identity (Byram et al., 2002). Some practical activities typically used in intercultural teaching are student exchanges, e-mails, project works, and films, allowing students to contact and interact with other cultures and experience cultural differences (Irimia, 2012; Constanza et al., 2018). Those activities are applied in language education and included in the teaching process to ensure that learners from different cultural backgrounds can be communicated smoother and non-conflicting (Willis and Willis, 2002; Larzén, 2005).

As practitioners of intercultural teaching, language teachers are not just those who teach knowledge about a foreign culture; but those who help learners gain a positive attitude toward people with different social identities in intercultural contexts. The ‘best’ language teacher is the person who can allow learners to understand relationships between their own and other cultures and acquire an interest in curiosity about ‘otherness’ and a critical awareness of themselves as well as others (Byram et al., 2002). For example, Breka and Petravić (2015) suggested foreign language teachers teaching in primary schools should cultivate their intercultural competence for the initial education and professional development. Freitas (2019) developed young learners’ attitudes of respect by conducting intercultural education in the second language teaching context.

Some researchers struggled to develop intercultural competence among language learners by implementing intercultural teaching in different contexts. For example, Cheng (2012) explored the intercultural competence and pedagogical practices of five Taiwanese EFL teachers through interviews, and the research revealed that pedagogical practices should reflect not only the interconnected world but also the local contexts and actual needs of students and teachers. Soomro et al. (2015) illustrated that cultural communicative teaching had not been attached enough attention in a large number of settings in Pakistan and Iran and suggested colleges and schools could make the integration between cultural awareness and language teaching processing. Others paid attention to developing native learners’ intercultural competence. Rehman and Umar (2019) conducted experimental research with 50 eighth-grade Pashtun students to examine the pragmatic intercultural influence on their reading comprehension. They suggested source and target cultures should be included in the English curriculum, and intercultural pragmatics should also be included in training programs. Vera and Ulrike-Marie (2016) implemented a problem-based intercultural learning unit in four secondary schools (grades 9–12) and varieties of teaching approaches (analytical/affective-experiential) and the language of instruction (German/English). The two approaches of analyzing film clips (analytical focus) and participating in simulation games (affective-experiential focus) can improve intercultural competence.

Some factors that affect intercultural competence have been explored in language education, such as language proficiency, intercultural awareness, and attitudes. For example, Piatkowska (2011) found that there was a close relationship between language proficiency and specific cultural awareness. Betty (2009) emphasized the importance of intercultural engagement in the curricula for native and international students. Güven (2015) investigated the Turkish university EFL learners’ attitude toward in-class learning of intercultural competence. The results revealed that the EFL learners’ intercultural competence might positively vary with attitude, motivations, and integration. Byram and Wagner (2018) argue a close relationship exists between the notion of culture, the language-culture nexus, and intercultural competence.

Based on previous research on intercultural competence, intercultural activities address linguistic competence education and intercultural teaching (Byram et al., 2002; Willis and Willis, 2002; Sharan and Elizabeth, 2016). A wealth of research on intercultural competence development is conducted in the field of language education, ranging from primary school to higher education, from the teachers’ attitudes to pedagogical practices, from the improvement of language proficiency to target culture instruction in the language curriculum (Yan, 2014). However, most of these studies are concerned with intercultural competence development of native language learners, while research on international students’ intercultural competence development is inadequate, especially in the Chinese context (Zhu and He, 2015; Zhu, 2017; Zhang, 2018; Yang et al., 2019).

This study centers on the research on intercultural competence development of international students in the Chinese context. It delves into the process of implementing intercultural competence within the context of teaching Mandarin to international students in China, exploring teachers’ perceptions of teaching intercultural competence in Mandarin language classrooms and revealing how intercultural activities are integrated into the classroom language teaching. The data types for this study include classroom observations, teachers’ interviews and reflective journals. To achieve the research purpose, the study addresses the following research questions:

(1) How do Mandarin teachers integrate intercultural activities into cultural teaching?
(2) How do Mandarin teachers integrate intercultural activities into language teaching?

## Methodology

### Participants

The present study was conducted in Mandarin courses for international students within Chinese universities. The sample included 5 Mandarin teachers with rich experience in teaching international students., aged 29–45. Of the total, 2 (40%) were lectures, 2 (40%) were associate professors, 1 (10%) was professor. Furthermore, the participants were recruited based on the following criteria: (1) having more than 1-year international student teaching experience, and (2) at least 6 months of overseas experience. The five Mandarin teachers’ personal information is listed below (see Table 1).

**TABLE 1:** Information of interviewees.

Name	Gender	Age	Professional title	Overseas experience	Mandarin teaching experience
Chai	Female	35	Lecturer	America (6 months); The Philippines (1.5 years)	1 year
Liu	Female	29	Lecturer	British (6 months); Singapore (1 year)	6 months
Wang	Male	39	Associate Professor	America (1 year) Britain (8 months)	3 years
Ye	Female	38	Associate Professor	Nepal (1 year) Korea (1year)	4 years
Zhou	Male	46	Professor	Holland (6 months) Australia (1 year)	2.5 years

### Data collection

Three types of data were collected in the study: classroom observation, teachers’ interviews and reflective journals. Most importantly, all procedures of data collection were permitted by every participant and consent forms were collected.

A classroom observation is watching a teacher’s performance in their classroom or learning environment. Classroom observation can effectively camera-record teacher behavior. According to Creswell (2003), “qualitative researchers seek to understand the context or setting of the participants through visiting this context and gathering information personally.” In this study, the researcher observed and videoed the five Mandarin teachers’ class activities in CSL teaching to obtain natural and first-hand materials about teachers’ classroom design concerning intercultural activities. When performing classroom observations, the researcher adopted the technique of narrative descriptions to document teachers’ teaching process and students’ reactions, especially intercultural activities; simultaneously, the video recordings of classroom performances were collected so that the researcher can further check the paper recordings and supplement more details by referring to video recordings. Altogether, the researcher conducted six 45-min classroom observations. The classes’ transcripts were checked and named as C1, C2, C3…… C6.

Moreover, to complement the findings of class observation, a semi-structured interview was utilized to explore teachers’ perceptions of teaching intercultural knowledge in Mandarin classes and what kind of teaching activities they prefer to choose, as well as how they handle the difficulties in developing intercultural competence in Mandarin teaching with the international students. The interview included four open-ended questions. In the first question, the participants were asked whether they had any difficulties with Mandarin teaching international students. The second question was addressed to participants to find out the restrictions in Mandarin teaching. The third question was posed to participants to determine how they deal with international students’ stereotypes and prejudices in class teaching. The last question was to explore how they handle intercultural education from their life experience. Interviews were carried out in Chinese with the selected five teachers face to face in their offices when they felt free. Each interview lasted 45 min, and the researcher asked for teachers’ permission to record the interview. Five recordings of interview were collected and then transcribed. Meanwhile, the researcher named the five interviews as T1, T2, T3, T4, and T5.

In addition, the participants were asked to keep a 4-week reflection journal about integrating intercultural activities into teaching Mandarin to international students and. In the reflective journal, the teacher narrated their teaching practices and students’ responses and expressed their views on intercultural teaching. Each teacher was asked to submit three reflection journals and fifteen reflective journals were collected and named J1, J2, J3, and J15.

### Data analysis

In qualitative research, a thick description is essential in presenting the original story and experience of the participants. Analysis of qualitative research starts when the data collecting begins and is an ongoing process (Creswell and Tashakkori, 2007). There are two main approaches for thematically analyzing qualitative data: inductive thematic analysis and deductive thematic analysis. Inductive thematic analysis is data-driven and themes are identified from the content of data; deductive thematic analysis is a top-down approach and the coding process is driven by some pre-existing theoretical concepts and ideas (Braun and Clarke, 2006). This study’s qualitative data were class observation, teachers’ semi-structured interviews, and reflective journals. The data were analyzed using inductive thematic analysis to identify themes relating to intercultural activities; simultaneously, deductive thematic analysis was adopted to explore aspects of intercultural competence development by referring to Byram’s (1997) intercultural competence framework.

The overall process of thematic analysis is as follows: First, the transcripts were analyzed using open coding. After completing open coding, the researcher categorized the intercultural activities in Mandarin teaching and identified the evidence of intercultural competence development by referring to Byram’s intercultural competence framework. Next, the main themes and sub-themes were determined related to the implementation of intercultural activities and the development of intercultural competence. The researcher and two foreign language teachers participated in the coding task. To increase the credibility of thematic analysis, they coded the same two materials together at first to understand and familiarize the coding method and framework before they began formal coding, respectively. After coding all the materials, the three coders compared, checked and discussed their coding results to reach consistency in interpreting and analyzing the data.

## Results

### Inductive thematic analysis

To investigate what and how intercultural activities were implemented in Mandarin language classroom, inductive thematic analysis was performed on the qualitative data and the results are shown in Table 2.

**TABLE 2:** Coding results of inductive thematic analysis of cross-cultural activities.

Themes	Subthemes	Examples
Topics for intercultural activities	Greeting in China	Have you eaten? 你吃了吗 ? (C1)
		Where are going? 你去哪儿 ? (C2)
	The celebration of the Spring Festival in China	Spring Festival Gala_‵_Reunion dinner (C4) Fireworks
	The celebration of moving to a new house	Red pocket, Fireworks, warm the pot 暧锅 (C3, C6)
	The traditional treatment of diseases in China	Herb_‵_*four diagnostic methods* 望,” “闻,” “问,” “切” (C2)
Methods of implementing intercultural activities	Double translation	Treat somebody a meal, please, Excuse me (请); Ok, walk,bank, line, career (行); (C1, C2, C3, C4, C5, C6)
	Comparison	Greetings in different countries; treating somebody in international students’ countries (C1, C4)
	Situation setting	Greetings; Go Dutch; Say sorry; Invite somebody (C1, C3, C6)
	Role play	Perform the roles of dialogue in textbook (C2, C4, C5, C6)

Through class observation, semi-structured interviews and the participant-teachers reflection journals, this study shows evidence of how Mandarin teachers involve intercultural activities in cultural teaching. It can be noted from Table 2 that Mandarin teachers attempt to present some culture-specific activities during their cultural teaching. For example, they introduce some typical Chinese cultural activities, such as how to greet each other, how to celebrate moving to a new house, how to celebrate the Spring Festival and how to see a doctor in China.

The methods teachers used to carry out intercultural activities include double translation, comparison, setting situations and role play (see Table 2). Firstly, double translation means the teachers use English as mediation for teaching Mandarin in the classroom. As the textbook’s content is written in Mandarin, the teachers explain it in English so the students can understand and translate it into their native languages. Secondly, the comparison activity is directly used to compare the similarities and differences between students’ cultures and other cultures. Thirdly, teachers apply the situation setting method to help students understand cultural diversity and how to deal with culture shock by encouraging students to imagine if they meet the situation and what they should do.

### Deductive thematic analysis

The study conducted deductive thematic analysis of the collected data to explore evidence of developing intercultural competence in CSL teaching using Byram’s intercultural competence model as coding framework which assumes intercultural competence comprised of attitudes, knowledge, skills of relating and interpreting, skills of discovery and interaction, and awareness. The results of deductive thematic analysis are presented in Table 3.

**TABLE 3:** Deductive thematic analysis on aspects of intercultural competence.

Themes	Description	Subthemes	Examples
Attitudes	Show an open attitude to different cultures and keep being curious about all the different cultures	Openness	Ask international students to show their own native language (T1,C2); Try to accept a foreign classmate to have dinner together (C3, T2, J5)
		Avoid stereotypes and prejudice	Ask international students to show their eating habit (T2, T5, C3, C4, J3, J6);
		Curiosity about other cultures	Enquire about how your classmates communicate with their parents (T3, C4)
Knowledge	Learning about social groups and cultures in one’s own country and other countries	Learning about greetings, festival, paying, etc. in Chinese culture	How to celebrate on moving to a new house in China (T1, C3, C6); How the traditional doctor treats your disease (T4, C2, J8)
		Learning about food, greetings, festivals, and verbal and non-verbal communication in other foreign cultures	Share one’s own culture to the class (T2, C5, J3)
		Learning about one’s own culture	Show and explain your own culture to five Chinese students on campus (T2, C5, J3)
Skills of relating and interpreting	Interpret a document or event from another culture	Make an explanation of events and behaviors in other countries	Share the latest news from one student’s country and ask the other students to interpret it (T4, C3, V1)
		Compare Chinese culture with one’s own culture	Compare ways of communication (T3,C1), clothing (T5, C3), food (T4, C4) in one’s own country and one’s own
Skills of discovery and interaction	Acquire new knowledge under the constraints of real-time communication and interaction	Learning new knowledge by communicating with classmates	Discussing with classmates on differences between Chinese culture and students’ own culture (T5, V4, J2)
		Interacting with local people	Make a Chinese friend, spend time with him/her, make observations, and write reflection journals (T4, C3, J5)
Critical cultural awareness	None	None	None

Table 3 shows intercultural competence development in five dimensions and indicates what aspects of intercultural competence are addressed by Mandarin language teachers in CSL classroom. As shown in Table 3, intercultural competence is developed in the following aspects: Firstly, intercultural attitude development includes having openness toward other cultures, avoiding stereotypes or prejudice, and showing curiosity about foreign cultures. Secondly, the improvement of knowledge dimension includes learning more about Chinese culture (e.g., food, greeting, festival, etc.), other foreign cultures (e.g., food, festivals, and verbal and non-verbal communication in India, Thailand, Indonesia and other countries), and learning about one’s own culture. Thirdly, the skills of interpreting and relating are fostered in understanding and explaining events and behaviors in other countries, and comparing Chinese culture with one’s own culture. Fourthly, the skills of discovery and interaction can be enhanced in learning new knowledge by communicating with classmates, and interacting with local people. However, there is no evidence of developing critical cultural awareness in Mandarin teachers’ CSL teaching.

## Discussion

According to Byram (2002), language teaching aims to help learners acquire linguistic competence when they need to communicate in speaking or writing. Moreover, it should also develop language learners’ intercultural competence, such as formulating what they want to say or write in correct and appropriate ways to talk with people from different cultures. Therefore, a language teacher should help learners improve their intercultural and linguistic competence. They should also help the language learners understand and accept people from other cultures as individuals with different distinctive perspectives, values, and behaviors and encourage the students to interact with people of other cultures in the language classroom (Deardorff, 2004; Zhang, 2007; Gu, 2016).

### Intercultural activities in Mandarin teaching

By analyzing the qualitative data, the study finds that teachers have a positive attitude toward Chinese cultural knowledge teaching to international students from different situations. Some evident cultural teaching examples can be seen in intercultural activities, such as the dialect of “儿” from Beijing culture, greetings in China, the celebration of Spring Festival in China, the celebration of moving to a new house, traditional Chinese clothes and the traditional treatment of diseases in China in the class observation. These activities are implemented using the methods of double translation, comparison, situations setting and role play.

#### Double translation

As international students are from different countries and have different native languages, they might not communicate with each other in their mother tongue. Therefore, English is the bridge language when the teacher teaches Mandarin in the classroom. In other words, since the textbook’s content is in Mandarin, the teacher is supposed to explain them in English first, and the students can receive the information in English. Still, they may translate English into their native languages to comprehend the knowledge and then ask and answer questions in Chinese or English.

**Example 1:** “请” (Please, treat, Excuse me)

*T: 请客, that means treat somebody. You can say person A请person B 客. For example, 今天我请客, that means today I will treat you.下次我请你的客, that means I will treat you next time.请坐, that means, please sit down. 请问that means Excuse me.我请你去我家, That means I invite you to my house.*

Example 1 shows the different usages of “请” in the phrases. Double translation was considered an exciting way of language processing for practicing Mandarin instead of simply translating from Mandarin to English or vice versa, which may be beneficial for arousing students’ intercultural interests in language learning by understanding some pragmatic functions as the content of translation between English and Mandarin.

#### Comparison

The comparison activity is directly used to compare the similarities and differences between students’ own culture and other cultures, as seen in Example 2. from the class observation that the teacher taught the greeting ways in China.

**Example 2:** Greetings in China

*T: As you know, “Have you eaten?” is a frequent greeting in China. When a Chinese asks you, “Have you eaten your dinner?” it does not refer to their want to treat you to dinner but as a usual greeting way as “Hello” or “How are you?”*

When teaching about the text of greetings, the teachers introduce the traditional Chinese ways of greeting and teach students how to do it in Mandarin. After preparing the Chinese greeting, the teacher asked the students to show their own countries’ greeting ways and encouraged other students to learn with them. After that, students formed into pairs to make presentations based on exploring the similar and diverse ways of greeting in different countries.

The purpose of the comparison activity is to enable students to understand their own culture better and acquire knowledge of other cultures, which is consistent with the knowledge part of Byram’s intercultural competence model (Byram et al., 2002). Based on their attitudes of openness and curiosity, students are interested in searching out knowledge that is different from their own culture.

#### Situations setting

To help students understand cultural diversity and how to deal with culture shock, the teachers always encourage students to imagine if they meet the situation and what they should do.

**Example 3**: AA 制 (Go Dutch)

*T: Different countries may have different ways of treatment. But in China, the difference between the treatment and Go Dutch. Imagine if your Chinese friend helped me a lot to pass your Mandarin examination, you want to show your gratitude to her by treating a dinner, you may pay all the bill, but if you go to the cinema with your cinema, you may pay the bill separately, that we called it AA制*

The teachers always set up different situations for the international students and asked them how to deal with them both in their own country and mainly taught them how to handle it to avoid cultural shock under the Chinese culture situation. Through this activity, teachers allow students to use Mandarin in different situations while experiencing cultural differences, which means students are motivated to practice their interactive skills and discover new knowledge when communicating with people from different cultural backgrounds like the teachers have to find the rule of starting conversations with “Have you eaten?” in China. In other words, it also means that students need to develop the ability to adapt, which indicates that one needs to quickly adjust to a new cultural environment and find the right way of communication and behavior (Deardorff, 2004). In addition, students began to relate these cultural differences to their own cultural beliefs, values, and behaviors. At the same time, they also accumulated knowledge of different cultures in the process of learning. Finally, openness to other cultures has always been the foundation of successful interactions (Byram, 2008; Díez-Bedmar and Byram, 2019). In general, using different intercultural situations enables students to behave and communicate appropriately in multicultural situations based on knowledge, skills, and attitudes (Deardorff, 2004).

#### Role play

Role play is an engaging activity for students and is commonly used in regular teaching. Mandarin classes are always designed to help international students understand the cultural differences between China and their own countries. For instance, to make the students master the dialogue from the textbook, the teacher always asked the students to perform the roles.

During the process, the students are required to use Mandarin phrases as much as possible. This way, knowledge of one’s own culture and other cultures can be acquired. Also, in this role play activity, language teachers need to encourage students to be open and curious about Chinese culture, which is consistent with Byram’s one of the standards of the “best teacher” (Byram et al., 2002). What is more, when imagining themselves as Chinese, students were able to be curious about Chinese culture and form ethnic relativism and empathy. In addition, as a person from a different country, evaluating the experience of Chinese culture from different perspectives is conducive to cultivating students’ critical cultural awareness (Byram, 2012).

Teachers use different methods such as double translation, comparing, situation setting and role play in cultural teaching, which may be implemented in different teaching stages. The double translation is the presentation stage in which the teacher presents students with language and cultural information, such as vocabulary lists, grammar points, or basic cultural knowledge. During the presentation stage, students passively receive information (Richards and Renandya, 2002; Sobkowiak, 2016). At the practice stage, the goal is to help students fully reinforce and internalize new knowledge, just like situation setting and comparing. Learners at the production stage can “integrate the newly learned language items into the previously learned ones on both receptive and productive levels” (Zhu, 2013). For instance, teachers employ role-play activities to encourage students to build new dialogues based on the textbook with the knowledge they have just learned and checked how much they have understood. Although the learners could master the required target cultural knowledge and skills through the different teaching activities which under the teacher’s close control to reinforce learners’ Chinese cultural usage, some problems still exist, such as the teacher’s inadequate input of cultural knowledge in advance, the unclear directions, and not leaving enough time for students to prepare.

### Intercultural competence development in Chinese as a Second language teaching

According to Byram et al. (2002), intercultural activities in language teaching aim to develop learners as intercultural speakers or mediators who can engage with complexity and multiple identities and avoid stereotyping.

#### Attitudes

Language teachers need to improve students’ attitudes, including certain characteristics such as curiosity, openness, and readiness to suspend disbelief about other cultures and beliefs about one’s native culture (Byram, 1997).

From the interview, we can happily find that the teacher participants always use different ways to evoke the language learners’ interest in learning about a different culture. According to Teacher Chai’s opinion:

*The main problems to make intercultural communication may be the lack of target language cultural knowledge and the regional problem. Therefore, she always uses the teaching methods to cope with those problems, such as (1) watching videos relating to culture (2) personal presentations about the students’ cultural experience (3) recommending some websites/books related to cultures (4) group brainstorming on some topics referring to cultures. (T1)*

As different people may have different perspectives on intercultural communication part, teacher Ye suggested in the interview:

*Stereotypes and prejudice are common in intercultural communication. I do not think it as an unsolved problem, but rather as the starting point for students to explore on them own. I always ask my students to do an assignment, coursework or presentation explaining such thing. It is actually very interesting to do so. (T4)*

Based on the class observation, semi-structured interviews and the participant-teachers reflection journal, the research coded the teachers’ teaching activities to develop students’ attitudes. It can be easily noted that the teachers tried every means such as making friends with the local people, sharing different country’s eating habits and enquiring about different countries communication styles to encourage international students have an open attitude to accept the different cultures, accept and avoid stereotype.

#### Knowledge

According to Byram (1997), intercultural knowledge means that individuals know their own and the interlocutor’s culture as they emerge in the course of social interaction. Except for the words or grammar teaching, the teacher recognized cultural teaching as an essential part of Mandarin teaching and designed some activities to help international students know more about cultures in the target language and their own cultures, as well as similar knowledge in other countries.

According to Byram (2005), intercultural dimensions in language teaching aim to develop learners as intercultural speakers or mediators who can engage with complexity and multiple identities and avoid the stereotyping that accompanies perceiving someone through a single identity. Therefore, as shown in Table 3, it is essential to note that Mandarin teachers try to improve students’ intercultural knowledge which refers to their own and the target language culture as they emerge in the course of social interaction. It is impressive that most participant-teachers used vivid examples to teach Chinese culture, such as the celebration of moving to a new house and the traditional Chinese treatments for diseases.

Meanwhile, the participant-teachers also ask international students to share their own culture with five local students on campus, which is beneficial to developing international students’ understanding of their own culture.

As a Mandarin teacher, Wang has taught native speakers in America for 1 year and he spent every period initiating “Chinese culture presentation” and drawing attention to encouraging students to experience Chinese culture vividly:

*The most way I used in the intercultural class is lecturing. I let the international students have some cultural experience. And then have some cultural lessons like paper cutting, making Chinese knots, doing some Chinese fans dance, playing the Tai Chi and writing calligraphy. (T3)*

This result is congruent with Irimia’s (2012) study that intercultural language teaching is the organic combination of language teaching and cultural teaching. It is significant to note that the interviewee respondents revealed that they consider the development of international students’ knowledge important in language teaching.

#### Skills of interpreting and relating

As a matter of fact, except intercultural knowledge, the other part of the skills cannot be neglected to make the language learners deal with the intercultural situation successfully. Skills of interpreting and relating deal with the ability to interpret events or documents from another culture, explain them, and relate them to events and documents from one’s own culture (Byram, 1997). Regarding the analysis, the participant teachers tried to give students opportunities to develop their skills of interpretation, as one of the participants shared the latest event from some students’ countries and asked the other students to express their interpretations and views on the event and gain a deeper understanding of the event through classroom discussion.

The teachers hold the importance of improving students’ skills of interpreting and relating. According to the teacher interviewees, they consider one person who has language ability cannot succeed in cross-cultural communication. Because interaction is the most of intercultural communication, which is expressed respectively by T1:

*Mastering language is not enough to have a successful cross-cultural communication. They do only communicate superficially, such as the daily greeting but cannot go further in communication which may cover the cultural aspects. I always encourage my students to explain the different behavior or the events in their own culture and compare them with other cultures. (T1)*

Similarly, from the point of Aguilar’s (2008) viewpoint, teaching language without culture is the best way to cultivate a fool with fluent spoken language. And language teachers should keep trying to improve the language learner’s interpreting and relating skills in class teaching.

#### Skills of discovery and interaction

There is no doubt that language learners are the main targets and participants of class education; developing their skills is the critical point (Welikala and Chris, 2008). According to this study, teachers try to encourage students to interact more with different countries and ask them to write a reflection journal about finding the differences among other countries.

From the interview of participant-teachers, they consider one person needs to discover the differences among different cultures and be eager to interact with the different cultures people:

*Language is a skill for people to communicate with others, but culture, is another thing. People may face the difficulties if they don’t communicate with the native speakers. So I would like to encourage my students to talk with local people. For example, I ask my students to make five interviews with local Chinese people and write the reflection journal to compare the differences between their own country and Chinese culture* (T2)

Similarly, a female teacher who has more than 4 years Mandarin teaching experience also holds an opinion about the importance of interaction:

*Cross-cultural communication includes so many aspects in different fields. I am afraid that only language ability cannot succeed in perfect communication and I believe language ability should have a cultural base to utilize appropriately. Asking my students to discover the differences among different countries is the main teaching method in my Mandarin classes.* (T4)

Therefore, language teachers, especially in higher education, need to encourage students to pay attention to the inter-culture and outer-culture (Wahyudi, 2012).

#### Critical cultural awareness

Byram (1997) holds that individuals need have a critical cultural awareness to evaluate ideas, products and practices in their own and other cultures from multicultural perspectives. Some researchers advocate that intercultural speakers must become aware of their own values and those of other countries (Byram, 2002; Wiseman, 2002; Tan, 2020). Whereas from the data analysis, most of the participant teachers rarely paid attention to improving international students’ critical cultural awareness, and most have no clear idea of how to operate it. Significantly, from the teacher-participants interview, some have a very vague perspective about developing students’ critical cultural awareness.

*Mostly, I always encourage my students to have an open attitude to accept the other cultures and they need to avoid stereotypes. If I ask my students to have a critical cultural awareness, it means they need to judge other cultures from your perspective. Therefore, I seldom highlight this point. (T2)*

From the interview, we find that the teacher participants also have the similar doubt to evoke the language learners’ critical cultural awareness of learning a different culture. According to Teacher Zhou’s opinion:

*I am keeping asking my students to avoid stereotypes and prejudice in intercultural communication. And I always offer great opportunities to students to interact with different countries people appropriately, if I ask them to keep a critical attitude towards the other cultures, they may feel puzzled and do not know how to deal with it. (T5)*

A similar result is acquired by Soomro et al. (2015), who suggested college level and schools could make an integration between cultural awareness and language teaching processing.

### The influence of intercultural activities on intercultural competence development

In Mandarin language classrooms, teachers have an awareness to improve the international students’ intercultural competence, design various cultural topics, and organize different forms of intercultural activities for students to practice their intercultural competence. Cultural knowledge, like other scientific knowledge, also has its scientific system. Language teachers should reasonably arrange different stages of cultural learning content, choose the suitable ways for students’ cognitive characteristics and the law of development of thinking, and follow the logical order to master the basic structure. The cultural part of classroom teaching design and the arrangement of the teaching activities should consider the effects of various factors on the learners (Yang and Li, 2017). It should also pay attention to the specific knowledge of the target language learning, and learners should also have a good understanding of the native language and the native culture and a positive attitude toward the target culture and other cultures.

The results of the deductive thematic analysis indicate that double translation, watching the videos, comparing, situation setting, and role-play are the effective and useful activities that the teacher adopts to improve the students’ intercultural competence. This is congruent with previous studies that language teachers attach importance to intercultural teaching in language classes (Cheng and Hong, 2014; Freitas, 2019). Learners’ intercultural awareness can be enhanced by comparing native cultures with the target culture and other cultures (Yu and Cao, 2015). For example, the activities of double translation regard English as the medium for instruction when the teacher teaches Mandarin in the classroom and the method is beneficial for improving students’ intercultural competence by adding cultural knowledge as the content of translation between English and Mandarin. It helps learners to acquire knowledge of culture- specify information and sociolinguistic awareness which are the distinctly important part for the students (Deardorff, 2004); especially, this method ensures the students correctly understand the content to be expressed and connect it with the target language and culture when introducing or translating a new word or phenomenon. During double translation, students can view their culture from a different perspective, which is conducive to promoting critical cultural awareness (Baker, 2016). Comparing is another activity that is directly used to compare the similarities and differences between students’ own culture and other cultures. This activity enables students to have a better understanding of their own culture and acquire knowledge of other cultures, improving students’ intercultural knowledge (Byram, 1997). After acquiring the knowledge and practice of their own culture, Chinese culture and other cultures concerning the same topic, students can discover new information and relate their own culture to others by comparing Chinese culture with those of other countries (Gu et al., 2010).

Intercultural activities in language teaching help learners understand and accept people from other cultures as individuals with different distinctive perspectives, values, and behaviors (Byram, 2002). To diversify classroom teaching activities, teachers should pay attention to the combination of didactic and experimental teaching methods, which can be arranged as lectures and explanations that focus on language and cultural knowledge, as well as role-playing, simulation activities, and visits that can cultivate language learners’ behavioral ability.

## Conclusion

The study explores the incorporation of intercultural competence in language education and investigates how Mandarin teachers facilitate international students’ intercultural competence. The findings suggest that integrating cultural topics closely related to real life and intercultural activities in the form of double translation, comparison situation and role play into Mandarin teaching and learning is beneficial to developing language learners’ intercultural competence, expanding their understanding of diverse cultures, developing their positive attitudes toward Chinese culture and others cultures, fostering their skills of comparing different cultures and skills of interacting among varying contexts, and increasing their critical cultural awareness.

The findings of the present study have the following implications. Firstly, integrating intercultural activities into Mandarin teaching has played an enlightening role in Chinese teaching. Secondly, it helps foreign language teachers have a new perspective from the combination of cultural and language teaching. Thirdly, it contributes to the innovation of international students teaching. Fourthly, it equips teachers and educators with the new dimensions to conduct relevant intercultural studies.

However, this study has some limitations. First, the subject of the study is limited, and more participants could yield more reliable findings to conduct the study. Second, this study paid attention to explore the perspectives of teachers and observers, but data from students’ perspectives were rarely collected (such as intercultural competence self-rating scale, student interviews, etc.). Third, the observation only lasted 4 weeks. Extending the time of the study would lead to in-depth and more detailed findings. Therefore, the results could be tentative for exploring the role of intercultural activities and their overall effects on developing learners’ intercultural competence.

## Data availability statement

The original contributions presented in this study are included in the article/supplementary material, further inquiries can be directed to the corresponding author.

## Ethics statement

Ethical review and approval was not required for the study on human participants in accordance with the local legislation and institutional requirements. Written informed consent from the patients/participants or patients/participants legal guardian/next of kin was not required to participate in this study in accordance with the national legislation and the institutional requirements.

## Author contributions

NL devised the project, the main conceptual ideas and proof outline, worked out almost all of the data collection and technical details, performed the data analysis, and contributed to the final version of the manuscript.
